# Hepatocellular carcinoma and antidepressants: a nationwide population-based study

**DOI:** 10.18632/oncotarget.12826

**Published:** 2016-10-23

**Authors:** Vincent Chin-Hung Chen, Chiao-Fan Lin, Yi-Hsuan Hsieh, Hsin-Yi Liang, Kuo-You Huang, Wei-Che Chiu, Yena Lee, Roger S. McIntyre, Hsiang-Lin Chan

**Affiliations:** ^1^ Department of Psychiatry, Chiayi Chang Gung Memorial Hospital, Chiayi, Taiwan; ^2^ Department of Psychiatry, Chang Gung University, Taoyuan, Taiwan; ^3^ Department of Child Psychiatry, Linkou Chang Gung Memorial Hospital, Taoyuan, Taiwan; ^4^ Department of Speech, Language Pathology and Audiology, Chung Shan Medical University, Taichung, Taiwan; ^5^ Department of Psychiatry, Cathay General Hospital, Taipei, Taiwan; ^6^ School of Medicine, Fu Jen Catholic University, Taipei, Taiwan; ^7^ Mood Disorders Psychopharmacology Unit, University Health Network, University of Toronto, Toronto, ON, USA; ^8^ Department of Psychiatry, University of Toronto, Toronto, ON, USA

**Keywords:** hepatocellular carcinoma, antidepressants, Taiwan national insurance

## Abstract

Hepatocellular carcinoma (HCC) is highly prevalent in Asia. Antidepressants have been associated with increase in hepatocellular carcinoma. This is the first Asian population-based study to evaluate the association between antidepressant use and risk of HCC. Based on Taiwans National Health Insurance Research Database, we conducted a nationwide population-based study. A total of 49,998 cases with HCC were identified and paired with 244,236 randomly selected controls. The data was analyzed via the conditional logistic regression model adjusting for several confounding factors. Use of tricyclic antidepressants (TCAs) and selective serotonin reuptake inhibitors (SSRIs) was associated with lower risk for HCC. No apparent association was found between use of other classes of antidepressants and HCC, including monoamine oxidase inhibitors (MAOIs), serotonin norepinephrine reuptake inhibitors (SNRIs), trazodone, mirtazapine and bupropion. The findings of a protective effect of TCAs and SSRIs for HCC should be interpreted with caution and warrants further research.

## INTRODUCTION

Hepatocellular carcinoma (HCC) is the second leading cause of cancer death worldwide including both high as well as low/middle income countries [1]. HCC is a fatal cancer and the 5-year survival rate for HCC is 25%-40% subsequent to diagnosis [2].

Antidepressant use is common and is increasing markedly in the past 2-3 decades [3,4]. Antidepressant offer symptomatic relief for disparate disorders, including anxiety disorders, depressive disorders, sleep disorders and pain disorders [5]. It is estimated that the prevalence of long-term antidepressant use (operationalized as longer than 24 months) has doubled in the US (3.0% to 6.9%) from 1999 to 2010 [6]. The high level of exposure of the general population to antidepressants invites the need for careful analysis of long-term safety.

Based on their mechanism of action, antidepressants can be divided into several classes including, but not limited to, the selective serotonin reuptake inhibitors (SSRIs), serotonin-norepinephrine reuptake inhibitors (SNRIs), tricyclic antidepressants (TCAs), and monoamine oxidase inhibitors (MAOIs). Most classes of antidepressants are biotransformed in the liver by isoenzymes of the cytochrome P450 system. In a review on antidepressant-induced liver injury, Voican el al. [7] concluded that all classes of antidepressants were associated with a risk of hepatotoxicity to varying degrees. Iproniazid, nefazodone, phenelzine, imipramine, amitriptyline, duloxetine, bupropion, trazodone, tianeptine, and agomelatine are most frequently reported to induce liver injury, while citalopram, escitalopram, paroxetine, and fluvoxamine are associated with a lower risk of hepatotoxicity [7]. Drug induced liver injury is common and is one of the most common reasons drugs are terminated during development and/or removed from the market after approval [8].

Furthermore, antidepressants have long been reported to be associated with the risk of developing several cancers, including HCC. For example, one clinical study indicated a transient but significant positive association between amitriptyline (a TCA) and HCC [9]. In another study, *in vitro* administration of paroxetine did not indicate genotoxic effects, but administration in male mice increased the incidence of tumor malignancy [10]. Moreover, animal studies assessing the carcinogenicity of sertraline (an SSRI) found a slight increase in benign liver tumor incidence in male mice [11]. A systematic review of the US Food and Drug Administration-approved registration data reported that duloxetine (SNRIs) was associated with increased incidence of HCC in female mice, and mirtazapine (NaSSA) was associated with increased incidence of HCC in male mice [12]. However, results have been inconsistent. One study reported that amitriptyline had an anti-cancer effect, promoting enhanced oxidative damage to cancer cells, including human hepatoma cells [13]. Two more studies also showed that fluoxetine and sertraline, both SSRIs, would induce apoptosis in human hepatoma cells [14, 15]. Additionally, one nationwide database record linkage study in Finland revealed that antidepressant use had no association with the incidence of liver cancer [16]. Lastly, Bendele et al. [17] administered fluoxetine to rats and mice and did not find an increase in the incidence of HCC.

The highest incidence rates of HCC in the world are reported in Asia and Africa [18]. In Taiwan, HCC has a particularly high incidence rate (32.97/100,000) and the major cause is chronic HBV infection, highlighting the importance of elucidating risk factors for its development [19]. Given the concurrent high rate (4.63%) of antidepressant use in Taiwan [20] and the extant conflicting evidence regarding an association with HCC development, we conducted a population-based nested case-control study to explore the associations between the use of antidepressants and HCC.

## MATERIALS AND METHODS

The National Health Insurance (NHI) program, the current health system in Taiwan, has been in effect since March 1, 1995. It is a compulsory social insurance paid by all residents and, as of December 2008, covered 99.5% of population [21]. The program's database, National Health Insurance Research Database (NHIRD), consists of registration files and original claim data for patient reimbursement. The data used in this study was obtained from NHIRD between January 1, 1997 and December 31, 2008, which provided detailed medical information about outpatient visits, hospital admission, prescription name and dose, medical procedures performed, and diagnostic codes.

To assess the association between antidepressant use and the incidence of HCC, we carried out a nationwide population-based nested case-control study. The outcome of the study was occurrence of HCC. We defined cases with HCC as two or more outpatient diagnoses or one inpatient diagnosis of HCC, and extracted diagnostic code information from NHI files according to the *International Classification of Diseases, 9th Revision, Clinical Modification* (ICD-9-CM). Each case with HCC diagnosis was linked to the Catastrophic Illness Claim Dataset to verify the diagnostic status again. The date of HCC claim was defined as the index date.

Five controls without a cancer diagnosis before the index date were randomly selected using incidence density sampling. The sampled date was the time of cancer cases diagnosed as HCC, defined as the index date. The controls were matched to cancer cases by year of birth, and individuals who were dead or discontinued the health insurance were excluded.

The pharmacological coding system we used to categorize antidepressants (N06A) is based on the Anatomical Therapeutic Chemical classification system [22]. We extracted prescription names and dose from the NHI database. All antidepressant use was identified and classified according to mechanism of action. This divided antidepressants into seven classes, including TCAs (i.e. amitriptyline, clomipramine, dothiepin, doxepin, imipramine, maprotiline, and melitracen), MAOIs (i.e. moclobemide, clorgyline, tranylcypromine, isocarboxacid, phenelzine, and selegiline), SSRIs (i.e. citalopram, escitalopram, fluoxetine, fluvoxamine, paroxetine, and sertraline), SNRIs (i.e., duloxetine, venlafaxine), serotonin antagonist and reuptake inhibitor (SARI) (i.e. trazodone), noradrenergic and specific serotonergic antidepressant (NaSSA) (i.e. mirtazapine), and norepinephrine dopamine reuptake inhibitor (NDRI) (i.e. bupropion).

Antidepressant exposure was standardized and the defined daily dose (DDD) according to WHO [22] was calculated. Cancer cases and controls with antidepressant use were categorized into four subgroups according to the DDDs (e.g. 28-83 DDD, 84-167 DDD, 168-335 DDD, and ≧336 DDD). Antidepressant use restricted to the year before cancer diagnosis was not included due to a potential protopathic effect [23].

Demographic variables including age at index date, income, and residential area were available in the NHI files. We performed a systematic review of the literature to identify factors that could confound our analyses, such as comorbid medical disorders and concomitant medication use. Comorbid medical disorders included depressive disorders, HBV infection, HCV infection, type 2 diabetes mellitus (DM), liver cirrhosis, and alcohol related disorders. Since NHI files didn't include smoking habit, we used chronic obstructive pulmonary disease (COPD) as proxy for smoking status. Concomitant medication use, including anti-HBV and anti-HCV medication, aspirin, non-aspirin NSAIDs, and statins, was controlled for.

### Statistical analysis

All statistical analyses were conducted using SAS version 9.2. [24] Descriptive statistics were performed to compare demographic features, comorbid disorders and concomitant medication use between HCC cases and controls.

We used conditional logistic regression models to explore our main interest, the effect of antidepressant on the risk of HCC. In order to clarify the impact of antidepressants, we surveyed different classes of antidepressants and different cumulative dose of antidepressants. A total of seven classes of antidepressants were included, and we divided the cumulative exposure into four dosage ranges, as mentioned above. The crude odds ratio (OR) and the adjusted OR were calculated, in each class of antidepressants and cumulative doses. P for trend was also calculated to clarify dose-dependent effects.

To compare the percentage of antidepressant use between cancer cases and controls, crude odds ratios were calculated. The corrected odds ratios were calculated with a 95% confidence interval (CI) after adjustment for demographic features and confounding factors including depressive disorders, HBV infection, HCV infection, type 2 DM, liver cirrhosis, alcohol related disorders, COPD, anti-HBV and anti-HCV medication, aspirin, non-aspirin NSAIDs, and statins. A *p* value of less than 0.05 was considered statistically significant.

### Ethics statement

The study was approved by the Institutional Review Board of Chiayi Chang Gung Memorial Hospital.

## RESULTS

We identified 49,998 HCC cases and 244,236 controls in NHI files between 1997 and 2008. The mean age of diagnosis of HCC was 57.41±13.36 years old, while the mean age of the controls was 57.29±13.27 years old. The age and sex of the controls were matched to cancer cases, so there was no significant difference between the two groups. Demographic features including age, income and urbanization are listed in Table 1. The levels of income and urbanization showed significant difference between cancer cases and controls (*p* < 0.001).

**Table 1:** Demographic data of cases and controls

		Cases		Controls		*p* value
		(*n*= 49998)		(*n*= 244236)		
Age						0.66
	≦40	9282	18.56 (%)	45302	18.55 (%)	
	41-50	14028	28.06 (%)	68910	28.21 (%)	
	51-60	11428	22.86 (%)	56210	23.01 (%)	
	61-70	9650	19.30 (%)	47011	19.25 (%)	
	71-80	4849	9.70 (%)	23213	9.50 (%)	
	≧80	761	1.52 (%)	3590	1.47 (%)	
Sex, Female		16258	32.52 (%)	79615	32.60 (%)	0.73
Income (NTD^a^)						<.0001
	0	7094	14.19 (%)	35955	14.72 (%)	
	1-25000	7136	14.27 (%)	37931	15.53 (%)	
	25001-40000	25815	51.63 (%)	107743	44.11 (%)	
	≧40001	9953	19.91 (%)	62607	25.63 (%)	
Urbanization^b^						<.0001
	Very high	12827	25.66 (%)	71539	29.29 (%)	
	High	23135	46.27 (%)	113407	46.43 (%)	
	Moderate	9508	19.02 (%)	40193	16.46 (%)	
	Low	4528	9.06 (%)	19097	7.82 (%)	

^a^ 1US $ = 32.3 New Taiwan Dollars (NTD) in year 2008

^b^ Quartiles by human development index

A comparison of the prevalence of comorbid medical disorders (e.g. depressive disorders, HBV infection, HCV infection, type 2 DM, liver cirrhosis, alcohol related disorders, and COPD) and concomitant medication use (e.g. anti-HBV and anti-HCV medication, aspirin, non-aspirin NSAIDs, and statins) are listed in Table 2.

**Table 2:** Medical diseases and drugs used with cases and controls

	Cases		Controls		*p* value

Medical diseases					
Depressive disorders^a^	2077	4.15 (%)	7326	3.00 (%)	<.0001
HBV infection^b^	6502	13.00 (%)	3883	1.59 (%)	<.0001
HCV infection^c^	6442	12.88 (%)	2789	1.14 (%)	<.0001
Type 2 DM	11042	22.08 (%)	28262	11.57 (%)	<.0001
Liver cirrhosis^d^	25134	50.27 (%)	23396	9.58 (%)	<.0001
Alcohol related disorders^e^	1105	2.21 (%)	945	0.39 (%)	<.0001
COPD	7088	14.18 (%)	27282	11.17 (%)	<.0001
Medications					
Anti-HBV,HCV	569	1.14 (%)	171	0.07 (%)	<.0001
Aspirin	6950	13.90 (%)	33882	13.87 (%)	0.87
Nonaspirin NSAIDs	30014	60.03 (%)	138062	56.53 (%)	<.0001
Statins	1858	3.72 (%)	15451	6.33 (%)	<.0001

COPD=chronic obstructive pulmonary disease (ICD-9: 490*-492*), Type 2 DM=type 2 diabetic mellitus (ICD-9: 249*, 250*, A181)

^a^ Depressive disorder (ICD-9: 296.2, 296.3, 300.4, 311).

^b^ HBV infection (ICD-9: 070.2, 070.3, V02.61)

^c^ HCV infection (ICD-9: 070.7, 070.41, 070.44, 070.51, 070.54, V02.62)

^d^ Liver cirrhosis (ICD-9: 571.2, 571.5, 571.6, 572.2, 572.3, 572.4, 572.8, or 573.0).

^e^ Alcohol related disorders (ICD-9: 291, 303.0, 303.9, 305.0, 571.0, 571.1, 571.2, or 571.3)

The overarching aim of our study was to compare the percentage of antidepressant use in cancer cases and controls. The results of the unadjusted conditional logistic regression; the stepwise conditional logistic regression after adjusting for demographic features, comorbid medical disorders, and concomitant medications use; and the *P* for trend are listed in Table 3. After adjustment for confounding factors, the use of TCAs (28-83 DDD [adjusted OR: 0.85, 95% CI: 0.78-0.93]; 84-167 DDD [adjusted OR: 0.80, 95% CI: 0.69-0.92]; 168-335 DDD [adjusted OR: 0.82, 95% CI: 0.69-0.98]; ≧336DDD [adjusted OR: 0.77, 95% CI: 0.62-0.96]) and SSRIs (28-83 DDD [adjusted OR: 0.82, 95% CI: 0.73-0.92]; 84-167 DDD [adjusted OR: 0.81, 95% CI: 0.69-0.97]; 168-335 DDD [adjusted OR: 0.77, 95% CI: 0.64-0.93]; ≧336DDD [adjusted OR: 0.61, 95% CI: 0.52-0.73]) were associated with lower risks for HCC than the control groups. The differences existed across 4 different cumulative dosages with dose-dependent effects. Among the remaining groups of antidepressants (e.g. MAOIs, SNRIs, SARI, NaSSA, NDRI), the risks for HCC had no significant difference with the control groups, except the subgroup with 84-167 DDD of SARI (adjusted OR: 0.76, 95% CI: 0.60-0.95).

**Table 3:** Associations of antidepressants use and hepatocellular cancer

Antidepressants	Cases (*n*= 49998)		Controls (*n*= 244236)		OR			
					Crude (95% CI^a^)	*P* for trend	Adjusted^b^ (95% CI)	*P* for trend
	*N*	%	N	%				
**TCAs**^c^						<.0001		<.0001
28-83DDD	1008	2.02	3740	1.53	1.32 (1.23-1.41)		0.85 (0.78-0.93)	
84-167DDD	368	0.74	1505	0.62	1.19 (1.06-1.34)		0.80 (0.69-0.92)	
168-335DDD	245	0.49	961	0.39	1.23 (1.07-1.42)		0.82 (0.69-0.98)	
≧336DDD	157	0.31	706	0.2	1.08 (0.91-1.29)		0.77 (0.62-0.96)	
**SSRIs**^e^						<.0001		<.0001
28-83DDD	618	1.24	2249	0.92	1.34 (1.23-1.47)		0.82 (0.73- 0.92)	
84-167DDD	288	0.58	1033	0.42	1.36 (1.20-1.55)		0.81 (0.69-0.97)	
168-335DDD	248	0.50	890	0.36	1.35 (1.17-1.56)		0.77 (0.64-0.93)	
≧336DDD	317	0.63	1401	0.57	1.10 (0.97-1.24)		0.61 (0.52-0.73)	
**MAOIs**^d^						<.0001		0.45
28-83DDD	797	1.59	3279	1.34	1.19 (1.10-1.28)		0.93 (0.85-1.03)	
84-167DDD	404	0.81	1590	0.65	1.24 (1.11-1.38)		0.92 (0.81-1.05)	
168-335DDD	446	0.89	1672	0.68	1.30 (1.17-1.44)		0.95 (0.83-1.08)	
≧336DDD	255	0.51	919	0.38	1.36 (1.18-1.56)		1.09 (0.92-1.29)	
**SNRIs**^f^						0.0001		0.49
28-83DDD	45	0.09	150	0.06	1.44 (1.03-2.00)		0.78 (0.49-1.22)	
84-167DDD	33	0.07	98	0.04	1.63 (1.10-2.42)		0.97 (0.57-1.63)	
168-335DDD	33	0.07	93	0.04	1.70 (1.15-2.54)		0.88 (0.53-1.48)	
≧336DDD	39	0.08	137	0.06	1.37 (0.96-1.96)		0.94 (0.59-1.50)	
**SARI**^g^**(Trazodone)**						<.0001		0.006
28-83DDD	471	0.94	1362	0.56	1.69 (1.52-1.87)		0.99 (0.87-1.14)	
84-167DDD	158	0.32	592	0.24	1.31 (1.10-1.56)		0.76 (0.60-0.95)	
168-335DDD	100	0.20	326	0.13	1.48 (1.18-1.86)		0.79 (0.60-1.06)	
≧336DDD	59	0.12	167	0.07	1.70 (1.27-2.29		0.77 (0.51-1.15)	
**NaSSA**^h^**(Mirtazapine)**						0.036		0.03
28-83DDD	22	0.04	87	0.04	1.17 (0.73-1.87)		0.58 (0.32-1.06)	
84-167DDD	14	0.03	35	0.01	1.93 (1.04-3.58)		0.54 (0.23-1.25)	
168-335DDD	7	0.01	30	0.01	1.13 (0.50-2.57)		0.65 (0.22-1.94)	
≧336DDD	9	0.02	25	0.01	1.66 (0.77-3.56)		0.61 (0.25-1.54)	
**NDRI**^i^**(Bupropion)**						0.023		0.78
28-83DDD	15	0.03	36	0.01	1.98 (1.09-3.63)		0.97 (0.43-2.23)	
84-167DDD	4	0.01	13	0.01	1.38 (0.45-4.25)		2.02 (0.40-10.2)	
168-335DDD	7	0.01	12	0.00	2.71 (1.06-6.88)		0.78 (0.23-2.69)	
≧336DDD	1	0.00	6	0.00	0.78 (0.09-6.48)		0.49 (0.03-7.64)	

^a^ 95% confidence interval

^b^ Adjusting with age, urbanization, income, depressive disorders, HBV infection, HCV infection, type 2 DM, liver cirrhosis, alcohol related disorders, COPD, aspirin, NSAIDs, statins

^c^ TCAs: tricyclic antidepressants

^d^ MAOIs: monoamine oxidase inhibitors

^e^ SSRIs: selective serotonin reuptake inhibitors

^f^ SNRIs: serotonin norepinephrine reuptake inhibitors

^g^ SARI: serotonin antagonist and reuptake inhibitor

^h^ NaSSA: noradrenergic and specific serotonergic antidepressant

^i^ NDRI: norepinephrine dopamine reuptake inhibitor

## DISCUSSION

To our best knowledge, this is the first Asian population-based study to evaluate the association between antidepressant use and risk of HCC development in the general population. After adjustment for confounding factors including comorbid medical disorders and concomitant medication, TCA and SSRI prescription was associated with lower risk for HCC development with dose-dependent effects. Use of other classes of antidepressants had no association with HCC development.

Previous studies based on human and animal data had suggested the potential hepatotoxic effects of antidepressants; however, our study did not show an increased risk for HCC across mechanistically dissimilar antidepressant exposures. Our finding was more similar with the nationwide database study in Finland which revealed that antidepressant use had no association with the risk of liver cancer [16]. In that study, antidepressants including SSRIs and non-SSRIs were assessed, though non-SSRIs were not classified into subgroups, and the number of liver cancer cases was much smaller (*n* = 415) than in our study [16].

Another finding in our study is that TCA and SSRI use was associated with lower risks for HCC. A lower risk of HCC associated with exposure to TCA and SSRI has not been previously reported in an Asian population. The magnitude of the lower risk identified herein requires replication and cautious interpretation. Our finding regarding lower risk with SSRIs replicates and extends evidence with SSRIs including fluoxetine and sertraline [14, 15]. Putative mechanistic pathways are suggested by Mun et al. [14] who reported that fluoxetine induced apoptosis of a HCC line via increased oxidative stress and reduction of mitochondrial membrane potential, while Chen et al. [15] reported that sertraline-induced apoptosis of HCC line is mediated via mitogen-activated protein kinase pathway.

According to a study conducted by Amerio et al., [12] preclinical data of antidepressant use including TCAs, SSRIs, SNRIs, trazodone, mirtazapine, and bupropion were reviewed. Non-SSRI antidepressants (i.e. duloxetine) and mirtazapine were suggested to be associated with HCC in an animal study. We also included non-SSRI antidepressants in our study; however, neither SNRIs nor mirtazapine were found to be associated with increased risk of HCC. These findings provided additional information regarding the safety profiles of non-SSRI antidepressants.

Previous epidemiological studies also explored the association between antidepressant use and cancer risk of other digestive system organs [16, 25, 26, 27, 28, 29]. One population-based study in the U.S. reported tricyclic antidepressant exposure was associated with increased trend of risk of esophageal adenocarcinoma [25]. No apparent association was found between antidepressant exposure and gastric cancer [16, 25, 26]. The aforementioned Finland study showed that antidepressant exposure had no association with pancreatic cancer [16]. Xu et al. [27] and Coogan et al. [28] both reported that SSRI exposure was associated with decreased risk for colorectal cancer. Studies on association between TCA exposure risk of colorectal cancer showed mixed and contradictory results. One study showed TCAs use had no association with the incidence of colorectal cancer [28], while the other study showed TCA use reduced colorectal cancer risk [29].

### Limitations and strengths

There are several methodological limitations that affect inferences and interpretations that can be made from our data. For example, we cannot assure accuracy in pathological biopsy diagnosis nor can there be assurances that recorded antidepressant prescriptions and their respective doses were adhered to. Moreover, there is no mechanism to determine whether antidepressants prescription is commensurate with antidepressant utilization. Moreover, the NHI files did not include records of other potential confounding factors including smoking and lifestyles which may be also related to the occurrence of HCC.

Notwithstanding the limitations, several strengths are inherent in this study. As the data were extracted from a nationwide database, the sample size was large and selection bias is minimized. The principal strength of this analysis is the perspective offered by the large database representative of the Taiwanese population.

## CONCLUSIONS

In conclusion, most classes of antidepressant use had a null association with the incidence of HCC. Our findings regarding the slightly protective effect of TCAs and SSRIs use should be interpreted cautiously, and warrant further investigation

## References

[1] Torre LA, Bray F, Siegel RL, Ferlay J, Lortet-Tieulent J, Jemal A (2015). Global cancer statistics, 2012. CA: a cancer journal for clinicians.

[2] Kim Y, Ejaz A, Tayal A, Spolverato G, Bridges JF, Anders RA, Pawlik TM (2014). Temporal trends in population-based death rates associated with chronic liver disease and liver cancer in the United States over the last 30 years. Cancer.

[3] Hemels ME, Koren G, Einarson TR (2002). Increased use of antidepressants in Canada: 1981-2000. The Annals of pharmacotherapy.

[4] Olfson M, Marcus SC (2009). National patterns in antidepressant medication treatment. Archives of general psychiatry.

[5] Noordam R, Aarts N, Verhamme KM, Sturkenboom MC, Stricker BH, Visser LE (2015). Prescription and indication trends of antidepressant drugs in the Netherlands between 1996 and 2012: a dynamic population-based study. European journal of clinical pharmacology.

[6] Mojtabai R, Olfson M (2014). National trends in long-term use of antidepressant medications: results from the U.S. National Health and Nutrition Examination Survey. The Journal of clinical psychiatry.

[7] Voican CS, Corruble E, Naveau S, Perlemuter G (2014). Antidepressant-induced liver injury: a review for clinicians. The American journal of psychiatry.

[8] McIntyre RS, Panjwani ZD, Nguyen HT, Woldeyohannes HO, Alsuwaidan M, Soczynska JK, Lourenco MT, Konarski JZ, Kennedy SH (2008). The hepatic safety profile of duloxetine: a review. Expert opinion on drug metabolism & toxicology.

[9] Selby JV, Friedman GD, Fireman BH (1989). Screening prescription drugs for possible carcinogenicity: eleven to fifteen years of follow-up. Cancer research.

[10] Kelvin AS, Mitchell ID, White DJ (1989). General and genetic toxicology of paroxetine. Acta psychiatrica Scandinavica Supplementum.

[11] Davies TS, Kluwe WM (1998). Preclinical toxicological evaluation of sertraline hydrochloride. Drug and chemical toxicology.

[12] Amerio A, Galvez JF, Odone A, Dalley SA, Ghaemi SN (2015). Carcinogenicity of psychotropic drugs: A systematic review of US Food and Drug Administration-required preclinical *in vivo* studies. The Australian and New Zealand journal of psychiatry.

[13] Cordero MD, Sanchez-Alcazar JA, Bautista-Ferrufino MR, Carmona-Lopez MI, Illanes M, Rios MJ, Garrido-Maraver J, Alcudia A, Navas P, de Miguel M (2010). Acute oxidant damage promoted on cancer cells by amitriptyline in comparison with some common chemotherapeutic drugs. Anti-cancer drugs.

[14] Mun AR, Lee SJ, Kim GB, Kang HS, Kim JS, Kim SJ (2013). Fluoxetine-induced apoptosis in hepatocellular carcinoma cells. Anticancer research.

[15] Chen S, Xuan J, Wan L, Lin H, Couch L, Mei N, Dobrovolsky VN, Guo L (2014). Sertraline, an antidepressant, induces apoptosis in hepatic cells through the mitogen-activated protein kinase pathway. Toxicological sciences.

[16] Haukka J, Sankila R, Klaukka T, Lonnqvist J, Niskanen L, Tanskanen A, Wahlbeck K, Tiihonen J (2010). Incidence of cancer and antidepressant medication: record linkage study. International journal of cancer.

[17] Bendele RA, Adams ER, Hoffman WP, Gries CL, Morton DM (1992). Carcinogenicity studies of fluoxetine hydrochloride in rats and mice. Cancer research.

[18] McGlynn KA, London WT (2011). The global epidemiology of hepatocellular carcinoma: present and future. Clinics in liver disease.

[19] Hung GY, Horng JL, Yen HJ, Lee CY, Lin LY (2015). Changing incidence patterns of hepatocellular carcinoma among age groups in Taiwan. Journal of hepatology.

[20] Wu CS, Shau WY, Chan HY, Lee YC, Lai YJ, Lai MS (2012). Utilization of antidepressants in Taiwan: a nationwide population-based survey from 2000 to 2009. Pharmacoepidemiology and drug safety.

[21] National Health Insurance Administration, Ministry of Health and Welfare (2013). Overview of National Health Insurance.

[22] World Health Organization Collaborating Centre for Drug Statistics Methodology (2013). ATC classification index with DDDs. WHO.

[23] Rothman KJ (1981). Induction and latent periods. American journal of epidemiology.

[24] SAS Institute: The SAS system for Windows (2011).

[25] Vaughan TL, Farrow DC, Hansten PD, Chow WH, Gammon MD, Risch HA, Stanford JL, Schoenberg JB, Mayne ST, Rotterdam H, Dubrow R, Ahsan H, West AB, Blot WJ, Fraumeni JF (1998). Risk of esophageal and gastric adenocarcinomas in relation to use of calcium channel blockers, asthma drugs, and other medications that promote gastroesophageal reflux. Cancer epidemiology, biomarkers & prevention.

[26] Hsieh YH, Chiu WC, Lin CF, Chan HL, Liang HY, Lee Y, McIntyre RS, Chen VC (2015). Antidepressants and Gastric Cancer: A Nationwide Population-Based Nested Case-Control Study. PloS one.

[27] Xu W, Tamim H, Shapiro S, Stang MR, Collet JP (2006). Use of antidepressants and risk of colorectal cancer: a nested case-control study. The Lancet Oncology.

[28] Coogan PF, Strom BL, Rosenberg L (2009). Antidepressant use and colorectal cancer risk. Pharmacoepidemiology and drug safety.

[29] Walker AJ, Card T, Bates TE, Muir K (2011). Tricyclic antidepressants and the incidence of certain cancers: a study using the GPRD. British journal of cancer.

